# Impact of Friedreich’s Ataxia on health-care resource utilization in the United Kingdom and Germany

**DOI:** 10.1186/1750-1172-8-38

**Published:** 2013-02-28

**Authors:** Paola Giunti, Julia Greenfield, Alison J Stevenson, Michael H Parkinson, Jodie L Hartmann, Ruediger Sandtmann, James Piercy, Jamie O’Hara, Leo Ruiz Casas, Fiona M Smith

**Affiliations:** 1 Department of Molecular Neuroscience, UCL Institute of Neurology, Queen Square, London, WC1N 3BG, UK; 2 Ataxia UK, 1-3 Brixton Road, London, UK; 3 Takeda Pharmaceuticals Europe Ltd, 61 Aldwych, London, UK; 4 Takeda Pharma GmbH, Aachen, Germany; 5 Adelphi Real World, Bollington, Cheshire, UK

**Keywords:** Ataxia, Friedreich’s Ataxia, Quality of life, Cost of illness, Resource utilization

## Abstract

**Background:**

Friedreich’s Ataxia (FRDA) is a neurodegenerative disorder that causes progressive damage to the central and peripheral nervous systems having a significant impact upon quality of life. With little information in the literature, cross-sectional observational studies were conducted in the UK and Germany to collect data on resource use and the burden of the disease on individuals and their caregivers.

**Methods:**

Cross-sectional observational studies were conducted in the UK and Germany to estimate the burden of FRDA on individuals and on the respective healthcare systems. A total of 75 individuals in the UK and 28 in Germany were recruited to the study. Participants in both countries were asked to complete a Patient and Caregiver Information Form (PCIF), regarding access to, and use of, healthcare resources, and the impact FRDA has on their lifestyle. In Germany, doctors were asked to complete a Patient Record Form (PRF). Analyses of annual direct and indirect resource utilization were conducted for both countries while costs were calculated for the UK only. These figures were compared to the costs associated with Parkinson’s disease; one of the most common neurodegenerative conditions and the one most similar in terms of disease progression.

**Results:**

The results showed that the annual burden of FRDA is significant and falls on the health and social care sectors, on society, on caregivers and on the individuals themselves. In the UK FRDA had a total annual cost per person of between £11,818 and £18,774 depending on whether the cost of long-term unemployment was included.

Typically the largest component of direct costs is associated with professional care. Given the high proportion of children and young adults recruited and the long disease duration, (typically 40-50 years for FRDA, compared with 20 years for Parkinson’s disease), these figures may underestimate the true burden of the disease.

**Conclusion:**

It is hoped that these estimates of resource utilization, can help in understanding the previously unquantified burden of FRDA. Given the long disease duration, management strategies should seek to minimise the impact of the condition on individuals and their caregivers, while maximising quality of life.

## Background

Friedreich’s Ataxia (FRDA) is an autosomal recessive neurodegenerative disorder that causes progressive damage to both the central and peripheral nervous systems. Although rare, it is the most frequent of the inherited ataxias, with an estimated prevalence in Europe of 1 in 50,000 [1]. FRDA is caused by mutations in the gene encoding the protein frataxin on chromosome 9q13, most commonly in the form of a GAA expansion within the first intron of the gene, which causes decreased production of frataxin [2]. Current evidence suggests that a loss of frataxin causes iron overload in mitochondria and an increase in free-radical production leading to oxidative damage and inactivation of mitochondrial respiratory chain enzymes, particularly complexes I, II, III and aconitase [3].

FRDA is characterized by progressive gait and limb ataxia, dysarthria, dysphagia, abnormal eye movements, hearing problems, sensory loss, muscular weakness, areflexia and extensor plantar responses. Non-neurological features include scoliosis, foot deformity, hypertrophic cardiomyopathy and diabetes mellitus [4]. Symptom onset typically occurs in childhood or adolescence, although late-onset cases are described. Life expectancy is around 40–50 years [3-5].

The management of individuals with FRDA requires a multidisciplinary approach. Most people diagnosed with the disorder require mobility aids such as a cane, walker or wheelchair by their teens or early 20s, and may require surgery for scoliosis or foot deformities [6]. Regular follow-up with annual echocardiograms and blood tests are necessary to detect and monitor the development of cardiac involvement and diabetes. Because of the progressive and disabling neurological and other features of the condition, FRDA has a significant impact upon quality of life [7].

Little or no data are available in the literature on the burden of FRDA on individuals with the condition, their families, caregivers, or society. More specifically, there is no record of previous investigations into resource utilisation by patients with FRDA. To address this gap the current questionnaire-based studies were conducted to collect information on the treatment and care of individuals with FRDA in the UK and Germany, and the burden of the disease on these individuals and their caregivers. Secondary objectives included a comprehensive description of people with FRDA (including their symptoms, diagnostic information and demographics), analysis of current management practices and treatment patterns.

Finally we compared the Cost of Illness (COI) of FRDA with Parkinson’s disease, one of the most common neurodegenerative conditions and one with similarities in terms of symptoms and progression of the disease [8].

## Methods

### Study design

Cross-sectional observational studies were conducted in the UK and Germany to estimate the burden of FRDA on individuals with the condition and on the respective healthcare systems. Participants in both countries were asked to complete a Patient and Caregiver Information Form (PCIF), regarding access to, and use of, healthcare resources, and the impact FRDA has on their lifestyle. The PCIF was jointly developed by Adelphi Real World (ARW) and Takeda with additional scientific input being provided by Takeda, Ataxia UK and a leading German Neurologist with a special interest in neurodegenerative and hereditary movement disorders. In addition, the PCIF was reviewed by a patient with FRDA in terms of the form’s ease of use, layout and font size, and the appropriateness of the questions/language used. All questions in the PCIF were closed questions.

For the purposes of this study, participants were considered to be children if aged 17 years and under, and adult if aged 18 years or over. In terms of who completed the PCIF, the parent or the child, children were encouraged to complete the form, with parental assistance if necessary. For very young children, the parent completed the form on their behalf. For individuals not capable of completing the PCIF, their caregivers were asked to complete selected sections of the questionnaire on their behalf.

Included in the PCIF were questions on patient demographics, FRDA medical history, healthcare resource utilization (hospitalizations, surgical procedures, consultation frequency, medications etc), loss of patient and caregiver productivity (Work and Productivity Impairment Index questionnaire – WPAI [9], the use of aids and adaptations, together with the impact FRDA has on their quality of life. In addition, patients were asked to provide details of the cost of any modifications made to their living accommodation, and how these modifications were paid for.

In the UK, individuals with the condition were recruited through the ataxia patient association, Ataxia UK, while in Germany, individuals were recruited by their physician. In the UK, Ataxia UK posted PCIFs to 200 patients randomly selected from Ataxia UK’s database, along with an introductory letter and patient information sheet explaining the study.

In Germany, a total of 25 neurologists were approached to take part in the study, of whom 5 agreed to participate. These 5 neurologists working in specialist centres in Göttingen, Munich, Freiburg, Bonn and Essen, were asked to complete a Patient Record Form (PRF), using information collected from the medical record, for individuals with FRDA who had been under their supervision for at least two years. Included in the physician completed PRFs were questions on patient demographics, consultation history, Friedreich’s Ataxia Rating Scale [10] score, activities of daily living, diagnostic tests, symptoms and medication history. Having completed the PRF, the neurologists were asked to post a corresponding PCIF, together with an instruction sheet, to each patient they included in the study so that the physician and patient-reported data could be linked. As in the UK study, no sensitive data were collected.

In Germany, information on direct medical resource use was provided by the physician as well as the participant. Information on diagnostic procedures was provided by the physician. Information on direct non-medical resources (such as aids and adaptations) was collected from participants only. Both physician and participants were asked to provide details on respite care. Where information was supplied by both the physician and participant, data were collected from the medical record as these were deemed more reliable than those from participant recollection. For both the UK and Germany, consent was implicit in the return of the surveys.

In terms of the recall period, with the exception of questions such as “had they ever had surgery” or “did they use mobility and/or other aids”, the recall period was 12 months. The recall period for work productivity was 7 days as this is the stated recall period for the Work Productivity and Activity Impairment (WPAI) tool.

The data were collated and analysed using STATA^®^ Version 10 (StataCorp LP, College Station, USA) and SPSS^®^ Version 15 (SPSS Inc., Chicago, USA). As sample sizes were low in both the UK (n=75) and Germany (n=28), qualitative (i.e. the reporting of frequencies and percentages) rather than quantitative statistics, were conducted.

Resource utilization can be analysed from a number of perspectives. In this analysis, annual direct and indirect resource utilization and costs were calculated for the UK only. For the German study, analyses were conducted on direct and indirect resource utilization.

In terms of resource utilization, the following were considered in the analysis:

• Patient visits to various Health Care Professionals (HCPs) (neurologists, primary care physicians (PCPs), physiotherapists, nurses etc.) in the previous 12 months.

• Individuals with FRDA hospitalized in the previous 12 months for medical or surgical procedures.

• Diagnostic tests performed on patients in the previous 12 months (Germany only).

• Current medication – prescription and non-prescription.

• Modifications made by individuals with FRDA to their living accommodation.

• Mobility aids used by individuals with FRDA.

• Short-term or respite care used by individuals with FRDA.

• Formal and informal caregiving provided by professionals and caregivers related to the patient.

• For patients currently in employment/education, work/teaching-time missed as a consequence of FRDA.

• Patients currently unemployed or on benefit.

### UK cost of illness

A bottom-up cost of illness methodology was employed; calculating costs on an individual basis before aggregating a total cost.

Costs were analysed in three main categories: (i) direct costs, (ii) costs associated with productivity and work loss and (iii) non-recurring (capital expenditure) costs. Direct costs were again divided into direct medical and other (non-treatment) direct costs. Direct medical costs included outpatient and community consultation costs, surgical and hospitalization costs, health-related service costs and medication costs, (including where appropriate, the cost of non-prescribed drugs). Other direct costs included social and respite care, and other non-treatment costs. Longer term costs such as mobility assistance and adaptations to living accommodation, realized over a number of years, were depreciated according to standard accountancy practices.

Unit cost data were obtained from published UK sources and applied to respondent estimates of resource use. Hospitalization costs were taken from the National Schedule of Reference Costs Year: '2008-09' - NHS Trusts Elective Inpatient HRG Data [11], while unit costs for outpatient consultations were taken from “Unit cost of health and social care” published by the PSSRU, University of Canterbury, UK 2009 [12]. Drug costs were sourced from the British National Formulary Number 59 [13].

Indirect costs included a loss of productivity associated with individuals with FRDA and their caregivers caused by absenteeism, an inability to work due to FRDA, and caregivers having to reduce their working hours.

A human capital approach was used to calculate productivity losses arising from reduced working time or caregiving whereby the mean time lost was multiplied by the average daily wage and by the number of working days per year (taken to be 220) in order to extrapolate to an annual cost. The average weekly income in the UK was taken to be £456 per week (Office for National Statistics, Labour Market Statistics, June 2010), which equates to an average daily income of £91.20 or £12.16 per hour assuming a 7.5 hour working day.

### Ethical approval

For the UK, approval was sought and obtained from Welwyn Clinical Pharmacology Ethics Committee to ensure the project met required ethical standards.

In Germany, ethical approval was obtained from the Ethical Committee of Göttingen or from the participating centre’s local ethics committees.

## Results

In the UK, questionnaires were sent to 200 individuals with FRDA. A total of 75 questionnaires were returned, with all 75 questionnaires being suitable for analysis; an overall response rate of 38%. In Germany, Neurologists at 5 centres (Gottingen, Munich, Freiburg, Bonn and Essen) agreed to participate in the study, providing information on a total of 28 individuals with FRDA.

### Demographic and socio-economic characteristics

The demographics and socio-economic characteristics of the study population are summarised in Table 1. The mean age of respondents was 32 ± 18 years (range 8-71) in the UK and 28 ± 14 years (range 8-56) in Germany. Sixty-seven percent of participants in the UK were adults aged 18 years and above, compared with 71% in Germany. Fifty-three percent in the UK and 61% in Germany lived with their parents while 35% and 34% respectively were in full time education. A higher proportion of female participants (52% in the UK and 64% in Germany) were recruited to the study. In Germany, the proportion of participants in full time employment was almost twice that of the UK (13% compared with 7%), while 30% of the study population were unemployed due to FRDA in the UK compared to 25% in Germany.

**Table 1:** Demographics and socioeconomic characteristics of participants with FRDA in the UK and Germany

	**United Kingdom**^**1**^	**Germany**^**2**^
Mean Age (years) ± SD	32 ± 18	28 ± 14
	(Range 8-71)	(Range 8-56)
	n=74	n=28
Gender	36 (48%) Male	10 (36%) Male
	39 (52%) Female	18 (64%) Female
	n=75	n=28
Adults (aged >18 years)	50 (67%)	20 (71%)
Children (aged <18 years)	25 (33%)	8 (29%)
	n=75	n=28
Mean age of symptom onset	14.1 years (±11.3 years)	11.3 years (± 6.1)
	Range 2-63 years	Range 4-28 years
	n=72	n=26
Time from symptoms to first doctor visit	1.6 years (± 3.1 years)	2.0 years (± 3.1)
	Range 0-19 years	Range 0-10 years
	n=63	n=27
Time from first doctor visit for symptoms to diagnosis	2.7 years (± 3.7 years)	1.9 years (± 2.3)
	Range 0-20 years	Range 0-8 years
	n=66	n=28
Living with their parents	40 (53%)	17 (61%)
	n=75	n=28
In primary/secondary/higher education	26 (35%)	11(34%)
	n=74	n=32*
Employment status (% employed full time)	5 (7%)	4 (13%)
	n=74	n=32*
Unemployed due to FRDA	22 (30%)	8 (25%)
	n=74	n=32*

^1^Source UK – PCIF.

^2^Source Germany – PCIF.

*Multiple responses.

### Symptoms

The mean age of symptom onset was 14.1 ± 11.3 years (range 2-63 years) and 11.3 ± 6.1 years (range 4-28 years) in the UK and Germany respectively. In the UK, 46% of individuals with FRDA first experienced symptoms as children aged up to 10 years, 39% in their teenage years and 10% aged between 21 and 30. In the UK, only 5% of respondents first experienced after their 30th birthday. In Germany, 43% first experienced FRDA-related symptoms as children aged up to 10 years, 43% as teenagers and 7% after their 21st birthday. (Note - 7% of individuals in Germany did not know when they first experienced symptoms of FRDA The mean time from symptom onset to first doctor visit was 1.6 ± 3.1 years (range 0-19 years) in the UK and 2.0 ± 3.1 years (range 0-10 years) in Germany. The time from first doctor visit to diagnosis was 2.7 ± 3.7 years (range 0-20 years) in the UK and 1.9 ± 2.3 years (range 0-8 years) in Germany.

With participants being asked to record all FRDA-related symptoms experienced over the previous 12 months, the results (Table 2) show participants experienced a range of symptoms linked to their condition including: stability and coordination problems, gait ataxia, scoliosis, foot and eye problems, fatigue, anxiety and depression, speech and swallowing difficulties, cardiac problems and diabetes.

**Table 2:** Participant reported symptoms experienced in the previous 12 months

**Symptoms**	**United Kingdom n(%)**^**1**^	**Germany n (%)**^**2**^
**Gait/Posture symptoms**		
Problems with lower limb coordination	73 (97%)	23 (82%)
Muscle weakness (upper or lower limbs)	68 (91%)	17 (61%)
Problems with upper limb coordination	70 (93%)	18 (64%)
Gait Ataxia (off balance when walking)	60 (80%)	18 (64%)
Body sway when standing	63 (84%)	13 (46%)
Problems with standing upright	60 (80%)	-
Muscle wasting (upper or lower limbs)	56 (75%)	17 (61%)
Problems with sitting upright	55 (73%)	-
Foot problems/deformities	50 (67%)	15 (54%)
Scoliosis (curved spine)	46 (46%)	20 (71%)
Problems with sitting or standing upright	-	23 (82%)
**Other Symptoms**		
Fatigue	65 (87%)	21 (75%)
Slowness and slurring of speech	62 (83%)	19 (67%)
Problems with swallowing	51 (68%)	0
Anxiety	39 (52%)	10 (35%)
Depression	37 (49%)	7 (25%)
Problems with eye movements	43 (57%)	8 (28%)
Palpitations	31 (41%)	6 (21%)
Heart enlargement	24 (32%)	9 (32%)
Other heart problems	15 (20%)	5 (17%)
Difficulty breathing	26 (35%)	6 (21%)
Loss of hearing	25 (33%)	6 (21%)
Loss of sight	22 (29%)	7 (25%)
Diabetes	9 (12%)	4 (14%)
Heart failure	5 (7%)	2 (7%)

^1^Source UK – PCIF.

^2^Source Germany - PCIF.

### Resource utilization in United Kingdom

In terms of health care resource utilization, a total of 17 UK participants (23%) had been hospitalized in the previous 12 months. Of those admitted, the mean number of admissions was 2.6 (range 1-10 admissions) and the mean length of stay was 9.3 days. Respondents were asked to provide the reason for the most recent admission. Of the 15 people who provided a reason, three people were admitted for cardiovascular reasons, and three for diagnostic examinations.

Respondents were also asked to state the reason for admission for any previous hospitalizations within the last 12 months. Of the 40 responses, the most common reasons for admission were cardioversion (12.5%), ECG / echocardiogram (7.5% each), and routine heart examination / stabilization of diabetes (5% each). In all, heart related admissions (not diabetes) accounted for 16 of the 40 admissions (40%).

In terms of surgical procedures, although none of the respondents had undergone foot surgery in the previous 12 months, 7 (9%) had had foot surgery at some time. One patient reported having had spinal surgery (on two separate occasions), while 10 (13%) reported having had spinal surgery at some time.

Respondents had an average of 14.12 consultations with health care professionals during the 12 month period; 5.09 consultations with doctors and 9.03 with other health care professionals. The most frequently consulted doctors were neurologists (61%), followed by cardiologists (57%) and PCPs (48%). The mean number of consultations over the same period was 4.37 for PCPs, but only 1.55 and 1.34 for cardiologists and neurologists respectively. With regards to other health care professionals, the majority of consultations occurred with physiotherapists, nurses and occupational therapists with an average of 7.77, 3.95 and 2.94 consultations per annum respectively (Table 3).

**Table 3:** Consultations with health care professionals in the previous 12 months

	**Consultations with HCPs in the UK n(%)**^**1**^	**UK** - **Mean number of consultations with HCPs**^**1**^	**Consultations with HCPs in Germany n(%)**^**2**^	**Germany** – **Mean number of consultations with HCPs**^**2**^
**Physicians**				
Neurologist	46 (61%)	1.34	17 (61%)	1.71
Cardiologist (routine)	43 (57%)	1.55	17 (61%)	0.75
PCP	36 (48%)	4.37	17 (61%)	2.61
Orthopaedic specialist	16 (21%)	1.37	13 (46%)	0.93
Paediatrician	17 (23%)	2.47	4 (14%)	0.54
Psychiatrist	5 (7%)	1.6	1 (4%)	0
**Other Healthcare Professionals**				
Occupational therapist	39 (52%)	2.94	10 (36%)	11.79
Physiotherapist	35 (47%)	7.77	23 (82%)	28.07
Nurse practitioner	21 (28%)	3.95	1 (4%)	0.14
Speech therapist	17 (23%)	2.76	7 (25%)	9.93
Pharmacist	9 (12%)	7.22	14 (50%)	3.43

UK and Germany Compared.

^1^Source UK – PCIF.

^2^Source Germany - PRF.

In terms of medications, 50% of responders took an average of 2.97 prescribed medicines and 21% purchased an average of 3.2 non-prescribed medications. Table 4 illustrates the most commonly prescribed and non-prescribed medications in the UK. Ten people (27%) were prescribed idebenone, and a further 2 people (14%) obtained it without prescription. Nine (24%) and 8 (57%) individuals respectively were either prescribed or purchased co-enzyme Q10, while a further 10 (27%) and 7 (50%) were either prescribed or purchased vitamin E. Other commonly prescribed prescription drugs including: beta-adrenoreceptor blocking agents, skeletal muscle relaxants and anti-epileptic drugs, were used by 24%, 19% and 16% of respondents respectively. Fish oils (omega 3 fatty acids) and other nutritional supplements, were used by 28% and 50% of respondents who purchased medications over the counter.

**Table 4:** **Most commonly prescribed and non**-**prescribed** (**including over the counter and other**) **drugs used by FRDA respondents in the UK**

	**Prescription drugs(n**=**37)**^**1**^	**Non**-**prescribed medication(n**=**14)**^**1**^
	**n(%)**	**n(%)**
Idebenone	10 (27%)	2 (14%)
Co-enzyme Q10	9 (24%)	8 (57%)
Vitamin E	10 (27%)	7 (50%)
Beta-adrenoreceptor blocking agents (Atenolol, Bisoprolol, Propranolol)	9 (24%)	0
Skeletal muscle relaxants	7 (19%)	0
Anti-epileptic drugs	6 (16%)	0
Fish oils (Omega 3 fatty acids)	0	4 (28%)
Other nutritional supplements (Multivitamins, minerals etc)	0	7 (50%)

^1^Source UK – PCIF.

Table 5 illustrates the number of adults and children taking idebenone and co-enzyme Q10. Of the 75 UK respondents (25 children and 50 adults), 10 received prescribed idebenone, of which, 9 (12%) were children. Of those purchasing idebenone without prescription, one was an adult (1.3%) and another was a child (1.3%). Of the 9 people (12%) prescribed co-enzyme Q10, 6 (8%) were children and of those purchasing the drug, 3 (4%) were children and 5 (6.7%) were adults.

**Table 5:** **Number of children and adult respondents taking Anti**-**Oxidants in the UK and Germany**

	**Prescription drugs(UK)**^**1**^		**Non**-**prescription(UK)**^**1**^		**Prescription drugs(Germany)**^**2**^		**Non**-**prescription(Germany)**^**3**^	
	**Child**	**Adult**	**Child**	**Adult**	**Child**	**Adult**	**Child**	**Adult**
Idebenone	9 (12%)	1 (1.3%)	1 (1.3%)	1 (1.3%)	5 (17.9%)	1 (3.6%)	3* (10.7%)	1* (3.6%)
Co-enzyme Q-10	6 (8%)	3 (4%)	3 (4%)	5 (6.7%)	0 (0%)	1 (3.6%)	0 (0%)	0 (0%)

*For the same patients, physician stated as prescription medication.

n=75 for the UK (25 children and 50 adults).

n=28 for Germany (8 children and 20 adults).

^1^Source UK – PCIF.

^2^Source Germany – PRF.

^3^Source Germany - PCIF.

In terms of social care, 19 respondents (25%) received formal care from a professional caregiver providing on average 34.15 hours of care each week. Three people with FRDA had experienced respite/short-term care in the previous 6 months (averaging a total of 2.67 admissions per person with a mean stay of 3.5 days). Twenty-six people (35%) were in primary, secondary or higher education, of whom 25 (21 children and 4 adults) required special educational support in the form of a classroom helper, a home tutor, a person to push their wheelchair or additional educational assistance.

Sixty-one percent of respondents had modified their living accommodation to ease the burden of FRDA. While extensive home improvements were the most expensive at an average of ₤33,166, the most common adaptations were changes to facilitate bathing at a mean cost of ₤5,133, and the provision of ramps at a mean cost of ₤2,564. The mean cost of other adaptations included: the widening of doors (£1,911), the installation of hoists (£1,684), electric beds (£1,758) and mattresses (£627), changes to the flooring (£1,561), stair rails (£65) and a stair lift (£1,197) (Table 6).

**Table 6:** **Total cost and funding source for home modifications and aids used by UK respondents**^**1**^

**Modification**	**Total cost(£)**	**NHS(£)**	**Social services(£)**	**Out**-**of**-**pocket(£)**	**Not stated**	**Mean cost of adaptation(£)**
Adaptation bath/ shower	189,927	12,219	105,760	55,305	16,643	5,133
		(6.4%)	(55.7%)	(29.1%)	(8.8%)	
Changes to home flooring	21,850	0	1,900	13,950	6,000	1,561
			(8.7%)	(63.8%)	(27.5%)	
Door Widening	34,408	1,775	17,431	7,426	7,776	1,911
		(5.2%)	(50.7%)	(21.6%)	(22.6%)	
Electric bed	33,407	13,264	5,350	9,550	5,243	1,758
		(39.7%)	(16.0%)	(28.6%)	(15.7%)	
Hand rail & Grab rail	1,277	290	697	0	290	256
		(22.7%)	(54.6%)		(22.7%)	
Hoist	32,000	5,600	25,600	0	800	1,684
		(17.5%)	(80%)		(2.5%)	
Ramps	76,933	4,433	49,967	18,000	4,533	2,564
		(5.8%)	(64.9%)	(23.4%)	(5.9%)	
Specialized Mattress	9,400	5,400	600	2,800	600	627
		(57.4%)	(6.4%)	(29.8%)	(6.4%)	
Stair Lift	5,985	1,500	995	1,500	0	1,197
		(37.5%)	(24.9%)	(37.5%)		
Stair rail	786	327	131	262	65	65
		(41.7%)	(16.7%)	(33.4%)	(8.3%)	
Extensive home improvement	199,000	0	143,000	11,000	45,000	33,166
			(71.9%)	(5.5%)	(22.6%)	

^1^Source UK – PCIF.

Funding for modifications to living accommodation and mobility aids can come from a number of sources. In the UK sources of funding included the National Health Service (NHS), Social Services, charitable grants and the respondents/families themselves. In terms of living accommodation, 37% of total costs were reimbursed by Social Services with 26% being paid for by the respondents themselves. Nine percent of costs were reimbursed by the NHS and 3% by charities. In contrast, the greatest proportion of costs related to mobility were reimbursed by the NHS (35%), 12% by Social Services, with respondents paying 33% themselves and charities 15%.

FRDA has an indirect cost that can be measured in an individual’s productivity at, and absenteeism from, work and the reduction in working hours required by the caregiver to help the person with FRDA. Thirteen percent of people with FRDA were in paid employment, working a mean of 23.6 hours per week. The mean time lost for those currently employed was 1 hour per week. For those attending classes, they spent an average of 24.8 hours per week in class, and missed an average of 3.9 hours. Seventy-seven percent of respondents required caregiver support. Eight of the 58 caregivers (14%) were professional caregivers. Twenty-nine percent and 19% of caregivers were in full or part-time employment. Twenty-two percent of caregivers had had to take time off work to care for the person with FRDA. On average, caregivers had a mean time off work of 26.15 hours per week.

### Resource utilization in Germany

In Germany, 13 participants, or 46%, had been hospitalized for FRDA-related symptoms in the previous 12 months. The mean length of stay was 5.2 days.

In terms of surgical procedures, while none of the respondents had undergone foot surgery in the previous 12 months, 21% had had surgery in the past. Overall 15% (n=27) had had spinal surgery at some time.

The majority of participants (61%) had consulted a neurologist in the previous 12 months, 61% a cardiologist, and 61% a PCP; seeing these doctors an average of 1.71, 0.75 and 2.61 times respectively. In terms of other health care professionals, the majority of consultations occurred with physiotherapists (82% of respondents), followed by occupational therapists (36%) and speech therapists (25%); seeing each an average of 28.07, 11.79 and 9.93 times respectively (Table 3).

Participants also provided details on health-related service utilization including the type of service, total cost and mode of reimbursement. Of the 28 participants, 6 (21%) reported using a health-related service. Eighty-two percent of respondents visited a physiotherapist at least once in the previous 12 months, but only 11% of patients provided details on reimbursement. In terms of health-related services, most were reimbursed by mandatory health insurance or paid for by the respondent. Of the 6 respondents who provided reimbursement details for chiropody, hippotherapy, physiotherapy and pilates, total costs equated to €1,445, of which 5% were paid for by mandatory health insurance, 28% by private health insurance and 67% by the respondent.

In terms of drug therapy, 64% of German participants took prescribed medication for FRDA-related symptoms, an average of 2.2 drugs per patient per annum. The most commonly prescribed drugs were idebenone (33%), beta-adrenoreceptor antagonists (22%), muscle relaxants (22%) and non-steroidal anti-inflammatory drugs (NSAID’s) (17%). Twenty-nine percent of participants reported utilizing non-prescribed medications; purchasing an average of 1.38 drugs for FRDA-related symptoms. The eight drugs bought without prescription were idebenone, Cranberola, Magnesium, L-carnitine, Cuprum Metallicum D6, Plumbum D6, Captobeta and Ranitidine.

Neurological testing was conducted at least once on all participants and included the Scale for the Assessment and Rating of Ataxia (SARA), the International Cooperative Ataxia Rating Scale (ICARS) and the Friedreich’s Ataxia Rating Scale (FARS). In addition, physicians conducted a number of diagnostic tests at regular intervals. These tests included: blood pressure, electrocardiograms, lipid profiles, echocardiograms, glucose tests, HbA1c, liver and kidney function tests, hearing and eye tests, spinal radiographs and MRI scans.

In terms of non-medical resource use, 7% of patients had used short-term respite care in the previous 12 months and of the 12 in an academic setting, 8 received special educational services. Sixteen participants (57%) had modified their accommodation to support their daily living. With a mean of 3.1 modifications per participant, the most common adaptations were modifications to the bath and shower facilities (63%), the fitting of handles/handrails (44%) and the installation of a lifter/hoist (31%) - (Table 7). With the source of funding for these adaptations being reported by the respondents themselves, Table 8 shows a total cost of €7,252 for home adaptations and aids excluding cars, (or €45,252 including the cost of cars), of which the majority was met through mandatory health insurance.

**Table 7:** **Modifications to living accommodation made by German respondents**^**1**^

		**Reimbursement sources for home adaptations/aids**			
**Modification (n)**	**Proportion**	**Mandatory health insurance n(%)**	**Private health insurance n(%)**	**Out of pocket n(%)**	**Not stated n(%)**
Adapted bath/shower (10)	63%	6 (60)	1 (10)	0	3 (30)
Bathroom alterations (4)	25%	3 (75)	0	0	1 (25)
Kitchen alterations (1)	6%	0	0	1 (100)	0
Adapted bed (4)	25%	3 (75)	0	0	1 (25)
Electric wheelchair (3)	19%	3 (100)	0	0	0
Handles/rails (7)	44%	4 (57)	0	1 (14)	2 (29)
Lifter/Hoist (5)	31%	5 (100)	0	0	0
Ramp (4)	25%	3 (75)	1 (25)	0	0
Speaking computer (1)	6%	1 (100)	0	0	0
Walking frame (1)	6%	1 (50)	0	0	0
Walking sticks (1)	6%	0	0	1 (100)	0
Wheel chair (3)	19%	2 (67)	0	0	1(33)
Other* (7)	44%	1 (14)	0	1 (14)	5 (72)

* Adaptation to house, folding chair, mattress, special seat, cars.

^1^Source Germany – PCIF.

**Table 8:** **Total cost and funding source for home modifications and aids used by German respondents**^**1**^

**Modification**	**Total cost(€)**	**Mandatory health insurance(€)**	**Private health insurance(€)**	**Out**-**of**-**pocket(€)**	**Not stated**
Adapted bath/shower	135	43	29	15	48
Alteration of bath room	436	182	0	182	73
Alteration of kitchen	364	0	0	364	0
Adapted bed	800	600	0	0	200
Electric wheelchair	2,400	2,400	0	0	0
Handles/rails	54	33	0	2	20
Lifter	800	800	0	0	0
Ramp	239	216	23	0	0
Speaking computer	364	364	0	0	0
Walking frame	240	120	0	0	0
Walking sticks	120	0	0	120	0
Wheelchair	1,200	800	0	0	400
Mattress	100	0	0	100	0
Cars	**38**,**000**	**0**	**0**	**0**	**38**,**000**
**Total(excluding cars)**	**45**,**252**	**5**,**557**	**52**	**902**	**38**,**741**
	**(7**,**252)**	**(5**,**557)**	**(52)**	**(902)**	**(741)**

^1^Source Germany – PCIF.

In terms of productivity lost in the previous 7 days, employed participants missed an average of 1.9 hours of work, while those in education missed an average of 5 hours of classes.

In terms of care, 60% of German participants required caregiver support to complete their daily activities. Fifty nine percent of all primary caregivers were the parents (and in particular the mother - 80%), 18% were professional carers, 18% partners and 6% other relatives. Caregivers *per se* provided a mean of 7 h of support each week of which the individual’s partners provided 7 h, the parent 4 h, other relatives 9 h and professional caregivers 16.7 h. Two caregivers reported having reduced their working hours by an average of 2.5 h per week in order to provide care.

### Annual cost of illness in United Kingdom

In terms of cost of illness (Table 9), the direct-medical costs incurred in caring for the 75 individuals with FRDA in the UK totaled £242,314 per annum. This figure, split between hospitalization costs (£50,067), consultation costs (£71,888), and medication costs (£120,359), gave a mean cost per respondent of £3,230. Non-direct medical costs of £322,940 per annum, were comprised of educational support costs (£14,688), respite care costs (£3,584) and professional caregiver costs (£304,668); averaging £4,306 per respondent. The total annual direct cost of caring for people with FRDA was therefore £565,254 (mean cost £7,537). Taking into account respondent reported expenses including living accommodation modifications (£44,268) and mobility aids (£57,419), the total annual direct non-medical cost of FRDA was £666,941.

**Table 9:** **Total annual direct medical and non**-**medical costs of FRDA in the United Kingdom** (**n**=**75**)

**Direct medical cost**	**Cost**	**Proportion of total direct medical cost**
Hospitalizations	£50,067	21%
Community / Outpatient Consultations	£71,888	30%
Prescription Medications	£83,456	34%
Non-prescription Medications	£36,903	15%
Total direct medical cost	£242,314	
**Direct non**-**medical cost**	**Cost**	**Proportion of Total Direct Non-Medical Cost**
Educational Support	£14,688	4.5%
Respite Care	£3,584	1.1%
Professional Caregiver Support	£304,668	94.3%
Total direct non-medical cost	£322,940	
**Direct non**-**medical cost including long term adaptation and mobility costs**	**Cost**	**Proportion of total direct non-medical costs**
Modification / Adaptation	£44,268	10.43%
Mobility	£57,419	13.52%
Total direct non-medical cost with long term costs	£424,627	
**Total direct cost excluding modification and mobility costs**	£565,254	
**Total direct cost including modification and mobility costs**	£666,941	

Annual indirect costs resulting from a loss of respondent and caregiver productivity were estimated at £4,426 and £214,989 respectively, a total of £219,415. Taking into account potential work loss caused by respondents being unable to work long-term, this estimated total cost rose to £741,079 (Table 10).

**Table 10:** Total annual indirect costs of FRDA in the United Kingdom (n = 50 adults)

	**Cost**
Respondent related costs (time off work)	£4,426
Respondent related costs (unable to work due to FRDA)	£521,664
Caregiver related costs	£214,989
**Total costs excluding unemployment due to FRDA**	£219,415
**Total costs including unemployment due to FRDA**	£741,079

Based on the responses received, FRDA resulted in a total annual cost of between £886,356 and £1,408,020 in the UK depending on whether the costs of long term unemployment due to FRDA (£521,664) were included or not (Tables 11 and 12). These equated to a cost per person of £11,818 and £18,774 respectively.

**Table 11:** **Total Cost of Friedreich**’**s Ataxia** (**including long term unemployment**) **in the United Kingdom (n = 75)**

	**Cost**	**Proportion of total cost**
Direct Treatment Costs	£242,314	17%
Direct Non-Treatment Costs	£424,627	30%
Work Loss^1^	£741,079	53%
Total Cost	£1,408,020	

^1^ Work Loss sample size = 50 adults.

**Table 12:** **Total cost of Friedreich**’**s Ataxia** (**excluding long term unemployment**) **in the United Kingdom (n = 75)**

	**Cost**	**Proportion of total cost**
Direct Treatment Costs	£242,314	27%
Non-Treatment Costs	£424,627	48%
Work Loss^1^	£219,415	25%
Total Cost	£886,356	

^1^ Work Loss sample size = 50 adults.

## Discussion

This study is the first to examine systematically the burden of FRDA in the UK and Germany, encompassing not only medical resource utilization, but also the burden on respondents in terms of symptoms, the impact on daily activities (including the need for mobility assistance and modifications to accommodation), and the requirement for caregiver support. The impact of caring for people with FRDA was also assessed.

The results show that the annual burden of FRDA is significant and falls on the health and social care sectors, on society due to work loss, on caregivers and on the individuals themselves. While the results of the UK COI study show a mean cost of health care per person of £3,230 per annum (including medications), this does not reflect the true burden of FRDA. When non-health care resource requirements (education, caregiver support *etc*) are included, the cost significantly rises to an estimated £7,537 per person. When indirect costs such as work loss, mobility aids and accommodation modifications are also taken into consideration, the estimated cost per person rises to between £11,818 and £18,774, depending on whether the costs of long-term unemployment or early retirement due to FRDA are included in the calculation. As direct health care costs represent such a small proportion of total costs, focussing on these and excluding the cost of long-term unemployment, would significantly underestimate the true burden to society. The main benefits of improved treatment options would be felt from a societal perspective should the need for caregiver support be reduced, if people were more able to participate in the workforce and if the need for modifications to living accommodation were reduced.

In terms of resource utilization, 61% of participants visited a neurologist in the UK and in Germany, while 47% and 82% visited a physiotherapist, and 48% and 61% a PCP, (UK and Germany respectively).

In Germany, the proportion of patients admitted to hospital for FRDA in the previous 12 months was twice that in the UK (46% versus 23%). Similarly, the proportion of participants who had had foot surgery at some time was more than double that in the UK (21% versus 9%), while non-prescribed drug use for FRDA-related symptoms was higher in the UK (3.2 versus 1.38 medications). The apparent difference in co-enzyme Q10 use between the two countries is notable with just one German patient taking co-enzyme Q10, compared with 17 (9 prescription and 8 OTC) in the UK. This difference may in part be a consequence of a high dose co-enzyme Q10/vitamin E trial having been conducted in the UK [14] and may also reflect a bias in the UK sample of patients. In general, patients willing to fill in questionnaires are more willing to participate in research and therefore more active in seeking therapeutic options.

Among the employed participants, mean work-time lost per patient was higher in Germany than in the UK (1.9 versus 1 hour per week), while the proportion of UK caregivers who had had to take time off work was almost twice that of Germany (22% versus 12%).

In interpreting these data, a number of caveats should be considered including the sample size. With 75 UK and 28 German patients taking part in the study, it is not possible to say whether they were representative of the FRDA populations although, considering the rarity of the condition, these sample sizes are considered reasonable.

The differing means of recruitment should also be taken into account. UK respondents could be more highly motivated (since they were known to the patient association and took the time to complete the questionnaire). While this is not a problem in itself, there is potential to introduce bias if these patients were atypical in terms of disease severity or in their ability to access health and social care resources.

Similarly the methodological differences between the two samples should be considered: whereas in the UK respondents were asked to complete PCIFs from memory, in Germany, in addition to the PCIFs completed by the patients, the recruiting physicians collected resource use and diagnostic information on PRFs. No such physician-reported, treatment-related data were collected in the UK study.

While patient recall is likely to be reasonable over the last 3 months, it may be less accurate (especially for routine events) as the recall period increases. Exceptions are major events such as hospitalizations and surgery, where there is evidence to suggest recall is as good over longer periods of time. While the recall periods specified in the questionnaires were designed to reflect this, an element of recall bias cannot be ruled out.

With no comprehensive studies in the literature, direct comparisons are difficult. For this reason we have chosen to compare the mean annual burden of FRDA with that of Parkinson’s disease, one of the most common neurodegenerative conditions and one with similarities in terms of symptoms and progression of the disease [8].

Findley et al reported a higher mean annual burden for advanced Parkinson’s disease of £28,700 [8], compared with between £11,818 and £18,774 for FRDA. Of the £28,700, £1,881 could be attributed to direct medical costs, £13,364 to direct non-medical costs and £12,454 to indirect informal care cost. Thirty-nine percent of advanced Parkinson’s disease patients were hospitalized in the previous 12 months, compared with 23% of individuals with FRDA in the UK and 46% in Germany. Parkinson’s disease patients experienced an average of 1.6 admissions and 2.9 consultations per year, while in the UK, individuals with FRDA experienced 2.6 admissions and 14.1 consultations. In terms of direct non-medical resources such as respite care, 13% of Parkinson’s disease patients had used respite care in the previous 12 months, compared with 5% of FRDA responders in the UK and 7% in Germany.

Parkinson’s disease severity is measured using the Hoehn and Yahr (H&Y) scale. All Parkinson’s disease patients, regardless of disease severity, can experience the OFF state. In the OFF state, individuals with Parkinson’s disease can experience tremors, stiffness, slowness of movement and/or periods of immobility. This can be due to medication wearing off, or a reaction to the medication itself, manifesting in a re-emergence of symptoms.

Sixty-eight percent of people with Parkinson’s disease reported in the study by Findley had a (H&Y) score of 3 or 4, spending between 0 and 25% of their waking time in an OFF state (=OFF 1). As expected, disease progression was associated with an increase in carer hours from 5.4 professional and 34 informal carer hours/week for OFF 1, to 21 professional and 51.25 informal care hours/week for OFF IV.

In contrast to Findley’s study, where the average age of individuals with Parkinson’s disease was 71.7 years, the average age of individuals with FRDA was significantly less at 32 years in the UK and 28 in Germany. With more children and young adults in the early to mid-stages of the disease, the cost of care is lower and may well be hidden (with younger parents, individuals with FRDA potentially have access to additional informal unpaid support from relatives, teachers and other caregivers). Given that Parkinson’s disease has a duration of 20 years whereas FRDA has a duration of 40 to 50 years, the cost to society may well be greater than for Parkinson’s disease.

In terms of future work, studies could include validating the PCIF for use in other countries and incorporating the use of the Friedreich’s Ataxia Impact Scale (FAIS) [15]. Data from the FAIS could then be compared to actual costs and burden of care to give an indication of the impact of the disease. Similarly, collecting data on clinical parameters such as the Friedreich’s Ataxia Rating Scale (FARS) may provide an indication of the cost and burden associated with disease severity.

## Conclusions

In conclusion it can be seen that the annual burden of FRDA is significant, falling on the health and social care sectors, on society (due to work loss), on caregivers, and on the individuals themselves. It is hoped that these estimates of resource utilization, from both a health sector and societal perspective, can help in understanding the previously unquantified burden of FRDA.

## Abbreviations

FRDA: Friedreich’s Ataxia; COI: Cost of Illness; PCIF: Patient and Caregiver Information Form; ARW: Adelphi Real World; WPAI: Work and Productivity Impairment Index questionnaire; PRF: Patient Record Form; HCP: Health Care Professional; PCP: Primary Care Physician; OTC: Over the counter; NHS: National Health Service; SARA: Scale for the Assessment and Rating of Ataxia; ICARS: International Cooperative Ataxia Rating Scale; FARS: Friedreich’s Ataxia Rating Scale.

## Competing interests

The authors declare that they have no competing interests. Dr Paola Giunti received no financial compensation for her work on the project.

## Authors’ contributions

PG – Reviewed the Patient and Caregivers Information Form and reviewed and revised the manuscript for intellectual content. JG – Was involved in the design of the Patient and Caregivers Information form and reviewed the manuscript. AS - Was involved in the design of the Patient and Caregivers Information form and reviewed the manuscript. MP - Reviewed the manuscript. JH – Was involved in the study design, the development of the Patient and Caregivers Information form, Patient Record Form, co-ordination of the project and in reviewing the manuscript. RS - Was involved in co-ordination of the project and in reviewing the manuscript. JP - Was involved in the study design and in reviewing the manuscript. JO’H – Was involved in analysis of the data and reviewing the manuscript. LRC - Was involved in analysis of the data and reviewing the manuscript. FMS - Was involved in the study design, the development of the Patient and Caregivers Information form, Patient Record Form, co-ordination of the project and in writing the manuscript. All authors read and approved the final manuscript.
